# Analysis of the association between millennium development goals 4 & 5 and the physician workforce across international economic strata

**DOI:** 10.1186/s12914-017-0126-2

**Published:** 2017-07-18

**Authors:** Christopher P. Morley, Dongliang Wang, Emily M. Mader, Kyle P. Plante, Lindsey N. Kingston, Azadeh Rabiei

**Affiliations:** 1 Department of Public Health and Preventive Medicine, SUNY Upstate Medical University, Syracuse, USA; 2 Department of Family Medicine, SUNY Upstate Medical University, Syracuse, USA; 3 Department of Psychiatry & Behavioral Sciences, SUNY Upstate Medical University, Syracuse, USA; 4 Center for Global Health & Translational Studies, SUNY Upstate Medical University, Syracuse, USA; 5 Cayuga Area Preferred/Plan, Ithaca, USA; 6 College of Medicine, SUNY Upstate Medical University, Syracuse, USA; 7 Department of History, Politics, and International Relations, Webster University, Webster Groves, USA; 8 St. Joseph’s Family Medicine Residency, Syracuse, USA

## Abstract

**Background:**

The Millennium Development Goals (MDGs) are 8 international development goals voluntarily adopted by 189 nations. The goals included health related aims to reduce the under-five child mortality rate by two-thirds (MDG4), and to reduce the maternal mortality ratio by three-quarters (MDG5). To assess the relationship between the healthcare workforce and MDGs 4–5, we examined the physician workforces of countries around the globe, in terms of the Physician Density Level (PDL, or number of physicians per 1000 population), and compared this rate across a number of years to several indicator variables specified as markers of progress towards MDG4 and MDG5.

**Methods:**

Data for each variable of interest were obtained from the World Bank’s Millennium Development Goals and World Development Indicators databases for 208 countries and territories from 2004 to 2014, representing a ten-year period for which the most information is available. We analyzed the relationships between MDG outcomes and PDL, controlling for national income levels and other covariates, using linear mixed model regression. Dependent variables were logarithmically transformed to meet assumptions necessary for multivariate analysis.

**Results:**

In unadjusted models, an increase of every one physician per 1000 population (one unit change in PDL) lowered the risk of not being vaccinated for measles-mumps-rubella (MMR) to 29.3% (*p* < 0.001, 95% CI: 22.2%–38.7%) and for not receiving diphtheria-tetanus-pertussis (DTaP) vaccination rate decreased to 38.5% (*p* < 0.001, 95% CI: 28.7% - 51.7%). Maternal mortality rate decreased to 76.6% (*p* < 0.001, 95% CI: 74.3% - 79.0%), neonatal mortality decreased to 58.8% (*p* < 0.001, 95% CI: 54.8% - 63.2%) and under-5 mortality rate decreased to 52.1% (*p* < 0.001, 95% CI: 48.0% - 56.4%), with every one-unit change in PDL. Adjusted models tended to reflect unadjusted risk assessments.

**Conclusion:**

The maintenance and improvement of the health workforce is a vital consideration when assessing how to achieve global development goals related to health outcomes.

## Background

The Millennium Development Goals (MDGs) are 8 international development goals that came out of the Millennium Summit of the United Nations in 2000 [1]. These goals were voluntarily adopted by 189 nations in an attempt to comprehensively address global poverty, hunger, maternal and child mortality, communicable disease, education, gender inequality, environmental sustainability, and a global partnership for development.

Maternal and child mortality is widely regarded as one of the best measures for the overall health and socioeconomic status of a country [2–4]. The fourth MDG (MDG4) aimed to reduce the under-five child mortality rate by two-thirds, and the fifth (MDG5) aimed to reduce the maternal mortality ratio by three-quarters. There has fortunately been global progress towards these goals. For example, the global under-five mortality rate has declined by more than half since 1990, from 12.7 million to about 6 million in 2015 [5]. The maternal mortality ratio has also been cut nearly in half since 1990, and globally more than 71% of births were assisted by a skilled health professional in 2014, which was an increase from 59% in 1990 [5]. However, progress has been uneven, with some countries achieving many goals while others achieved very few, if any, of the established targets. Tracking of progress under the MDGs has also been impeded by gaps, discrepancies, and reporting delays in data [6].

The *United Nations Millennium Development Goals Task Force Reports on Child Health and Maternal Health* declared that one of the largest barriers to providing interventions and achieving MDGs was the lack of properly trained healthcare providers distributed across the globe [5]. The WHO estimates that healthcare systems comprised of fewer than 23 healthcare workers per 10,000 population are unable to properly deliver necessary health services to a given region [7]. Several studies from across the world have suggested that an increase in physician or skilled healthcare worker density can produce a significant reduction in maternal and child mortality rates [8–10], and other studies have demonstrated the relationship between physician density and HIV/AIDS prevalence [11]. However, healthcare improvement in developing nations is often handled in response to distinct crises, or with internationally-based non-governmental agencies (NGOs) providing distinct and specific services. Additionally, as Chen [12] and others [7] have pointed out, the development of a health workforce “takes time, at least a decade and often a generation” [12].

To assess the relationship between the healthcare workforce and MDGs 4–5, we examined the physician workforces of countries around the globe, in terms of the Physician Density Level (PDL, or number of physicians per 1000 population), and compared this rate across a number of years to several indicator variables specified as markers of progress towards MDG4 and MDG5.

## Methods

### Data selection

Data for each of the variables of interest were obtained from the World Bank’s Millennium Development Goals [1] and World Development Indicators databases [13] for 208 countries and territories from 2004 to 2014, which represents the ten-year period for which the most information is available. We elected to utilize all nations and territories for which data were available, regardless of whether each of the territories were one of those who formally adopted the MDGs. Variables identified as MDG outcome measures exist for each country, regardless of whether the data were collected as part of MDG assessment, or as part of more general knowledge gathering. We therefore measured the effect of PDL upon outcomes identified under the MDG framework as important, regardless of whether a specific country or territory actually adopted MDGs.

Given the sparsity of data, we had to rely on the best, most complete indicator variables available at the time the study was initiated. Although a variety of healthcare workforce variables may be conceptualized as having an impact upon health outcomes, such as the number of nurses or community healthcare workers in a given population, the most consistently reported rates were for PDL. We therefore utilized this rate as the key independent variable in our analyses. In addition to the key variable of interest (the PDL), we were able to acquire data for several covariates, including country income level, female literacy rate, gross domestic product (GDP) per capita, and percent urban population, from the World Bank Development Indicators [13].

Our outcome variables were informed by the specified UN Development Goals. For MDG4, the key indicators specified are the under-five mortality rate per country (MDG4.1), the infant mortality rate (MDG4.2), and the proportion of 1 year old children immunized against measles-mumps-rubella (MMR) (MDG4.3). Data were available for each of these indicators, as well as for diphtheria-tetanus-pertussis (DTaP) vaccination. Unfortunately, we found data for maternal outcomes to be much more sparse; however, maternal-mortality data (MDG5.1) were available for many nations across a broad study period, from 2004 to 2014. The outcome variables of interest for these analyses therefore included MMR vaccination coverage, DTaP vaccination coverage, the estimated maternal mortality rate, neonatal mortality rate, and mortality rate for children under five for each country and territory included in analysis.

Several of the countries within our sample lacked consistent measurement of PDL, with some countries only reporting two or three measurements across our study years. However, estimations revealed little within-country variance for PDL; therefore, we calculated the average PDL from 2004 to 2014. Countries were further divided into four categories based on the average PDL across the sample: low (0 to 0.2 per 1000 population), lower middle (0.2 to 1.2 per 1000 population), upper middle (1.2 to 2 per 1000 population), and high (2 to 5 per 1000 population).

A total of 2496 data points (208 countries and territories × 12 years) were available for analysis. However, there were more than 6 physicians per 1000 populations in only three countries (San Marino. 6.5; Spain, 7.05; Timor-Leste, 8.4). These countries were very influential to the regression results; we therefore ran models which excluded these three countries from analysis, as well as inclusive models that incorporated these outliers.

### Analysis

The negative skewness of the vaccination coverage rate variables, and positive skewness of the mortality variables, required that each dependent variable be transformed to meet the assumptions necessary for multivariate analysis. The vaccination coverage rate variables were inversed to create a positive skew, changing the interpretation to be the rate of children not vaccinated. We calculated the natural log of all dependent variables, resulting in the estimation of relative risks in subsequent analyses.

The relationship of the PDL with each of the five outcomes was graphically described. The curves suggested assessing the impact of the physician density level over the five outcome variables using linear mixed models, which is equivalent to Poisson regression. The time (year) and its interaction with the PDL were included as fixed effects and the country was included as a random effect. A first-order autoregressive structure was used to model the correlations between observations at the different years from the same country, which is associated with the smallest Akaike information criterion (AIC) as compared to simple, compound-symmetry, Toeplitz, and other correlation structures. The models were fitted using the restricted maximum likelihood method and the relative risks and odds ratios (with *p*-values and 95% confidence intervals) were reported. No additional covariates were included for the unadjusted RRs and ORs. For the adjusted RRs and ORs, a fixed set of potential confounders (i.e., GDP per capita, female literacy rate, percent urban population) were considered in the model. Visual inspection of residual plots did not reveal any obvious deviations from homoscedasticity or normality. All statistical analyses were conducted in SAS 9.3 (SAS Institute, Cary, NC); mixed effects models were conducted using PROC MIXED.

## Results

**Fig. 1:**
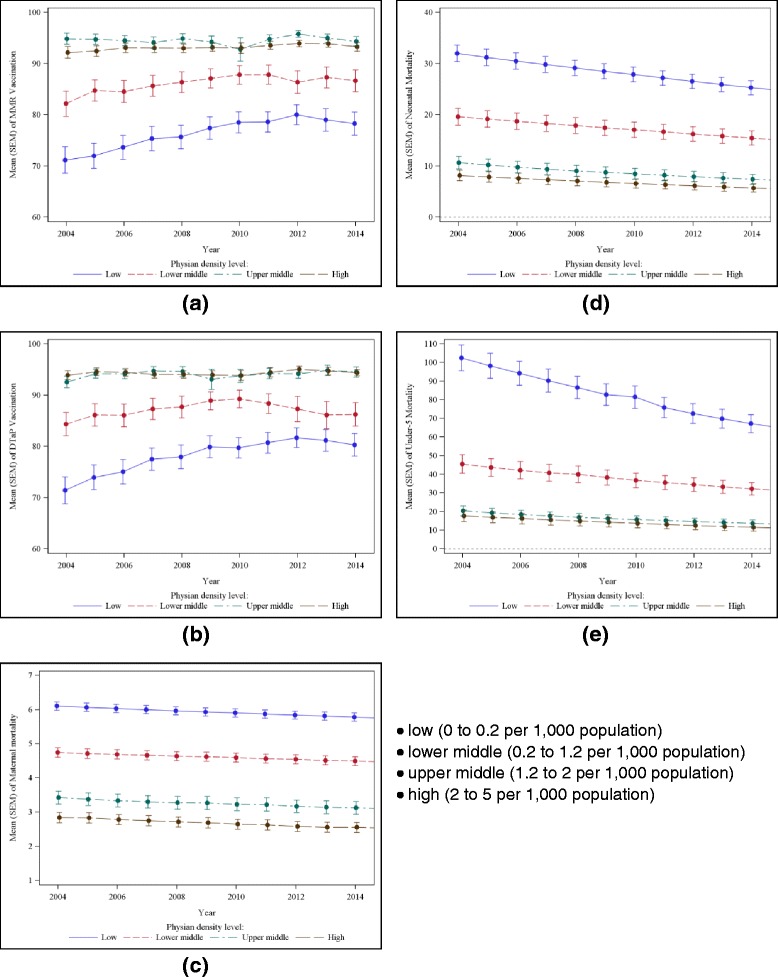
The relationship of the physician density level with each of the five outcomes from 2004 to 2014. Countries were divided into four categories based on the average physician density level across the sample: • low (0 to 0.2 per 1000 population); • lower middle (0.2 to 1.2 per 1000 population); • upper middle (1.2 to 2 per 1000 population); • high (2 to 5 per 1000 population). **a** Mean MMR Vaccination Rate. **b** Mean DTaP Rate. **c** Mean Maternal Mortality Rate. **d** Mean Neonatal Mortality Rate. **e** Mean Under-5 Mortality Rate

Figure 1 shows the relationship of the PDL with each of the five outcomes. Figure 1a and b show that MMR and DTaP vaccination rates increased as physician level increased when the PDL was less than 2. When there were more than 2 physicians per 1000 population, the PDL had no impact on the MMR and DTaP vaccination rate. Therefore, the regressions for the MMR and DTaP vaccination rates were only performed over countries having 2 or fewer physicians per 1000 population. These results are provided in Table 1. With a one-unit increase in the PDL, the risk of not being vaccinated for MMR decreased to 29.3% (*p* < 0.001, 95% CI: 22.2%–38.7%); and the risk of not being vaccinated for DTaP decreased to 38.5% (*p* < 0.001, 95% CI: 28.7% - 51.7%) for the unadjusted models.

**Table 1:** Impact of physician density level on targeted outcomes, 205 countries and territories excluding San Marino, Spain and Timor-Leste, 2004–2014, unadjusted and adjusted mixed effect models results

Outcome variable	Poisson regression			
	Unadjusted		Adjusted^a^	
	Relative risk (*p*-value)	Confidence interval	Relative risk (*p*-value)	Confidence interval
MMR Un-Vaccination Rate^b^	0.293 (<0.0001)	0.222–0.387	0.346 (0.0002)	0.200–0.600
DTaP Un-Vaccination Rate^b^	0.385 (<0.0001)	0.287–0.517	0.373 (0.0009)	0.211–0.659
Neonatal Mortality Rate	0.588 (<0.001)	0.548–0.632	0.753 (<0.0001)	0.688–0.824
Under-5 Mortality Rate	0.521 (<0.0001)	0.480–0.564	0.675 (<0.0001)	0.610–0.747
Maternal Mortality Rate	0.766 (<0.0001)	0.743–0.790	0.858 (<0.0001)	0.823–0.894

^a^The confounding effects of country income level, female literacy rate, gross domestic product (GDP) per capita, and percent urban population were adjusted

^b^The regression was performed over all countries with the average number of physicians per 1000 population less than or equal to 2 from 2004 to 2014

Figure 1c-d show that maternal mortality, neonatal mortality, and under-5 mortality rates decreased as PDL increased. In the unadjusted models, a one-unit increase in the PDL, the risk of maternal mortality decreased to 76.6% (*p* < 0.001, 95% CI: 74.3% - 79.0%); risk of neonatal mortality decreased to 58.8% (*p* < 0.001, 95% CI: 54.8% - 63.2%); and risk of under-5 mortality decreased to 52.1% (*p* < 0.001, 95% CI: 48.0% - 56.4%).

The results from the fully adjusted mixed effects models are provided in the right panel of Table 1. PDL was still a statistically significant predictor of all outcome variables. For a one-unit increase in the PDL of a country, the risk of not being vaccinated for MMR decreased to 34.6% (*p* = 0.0002, 95% CI: 20.0% - 60.0%); the risk of not being vaccinated for DTaP decreased to 37.3% (*p* = 0.0009, 95% CI: 21.1% - 65.9%); the risk of maternal mortality decreased to 85.8% (*p* < 0.0001, 95% CI: 82.3% - 89.4%); the risk of neonatal mortality decreased to 75.3% (*p* < 0.0001, 95% CI: 68.6% - 82.4%); and the risk of mortality for children under 5 decreased to 67.5% (*p* < 0.0001, 95% CI: 61.0% - 74.7%).

The analysis results using all 208 countries were provided in Table 2. Without considering any other covariates, the PDL still showed significant impact over each of the five outcome variables. With the outliers included and adjusting for covariates, PDL’s impact on the MMR and DTaP vaccination was not significant; however, its impact on the maternal, neonatal and under-5 mortality rate remained significant. All of the relative risks in Table 2 were larger than the counterparts in Table 1.

**Table 2:** Impact of physician density level on targeted outcomes, 208 countries and territories, 2004–2014, unadjusted and adjusted mixed effect models results

Outcome variable	Poisson regression			
	Unadjusted		Adjusted^a^	
	Relative risk (*p*-value)	Confidence interval	Relative risk (*p*-value)	Confidence interval
MMR Un-Vaccination Rate	0.691 (<0.0001)	0.625–0.764	0.875 (0.1729)	0.723–1.059
DTaP Un-Vaccination Rate	0.713 (<0.0001)	0.644–0.789	0.905 (0.2955)	0.751–1.091
Neonatal Mortality Rate	0.655 (<0.0001)	0.611–0.703	0.814 (<0.0001)	0.756–0.876
Under-5 Mortality Rate	0.597 (<0.0001)	0.551–0.647	0.762 (<0.0001)	0.701–0.829
Maternal Mortality Rate	0.813 (<0.0001)	0.786–0.840	0.813 (<0.0001)	0.786–0.840

^a^The confounding effects of country income level, female literacy rate, gross domestic product (GDP) per capita, and percent urban population were adjusted

## Discussion

The density of physicians in low and middle-income countries appears to have been an important factor in observed progress toward health-related MDGs. While this may seem to be an overly obvious finding, strategies aimed at maintaining or increasing the number of physicians living and practicing, on a permanent basis, in developing nations are not often at the forefront of conversations about international development, which often focuses upon bringing aid (in the form of supplies and workers) to the developing nation on a temporary, often crisis-driven basis.

Strategies to train a physician workforce in the developing world, and to encourage physicians to stay in-place and practice in the developing world, are likely difficult to achieve, as “brain drain” from developing countries is a distinct problem across all highly educated professions [14]. Of course, migration patterns follow international economic trends – it will likely take an internationally coordinated effort to create incentives for physicians (and other healthcare workers as well) to stay in place.

There are, of course, other realities to consider, regarding the delivery of healthcare in resource poor parts of the world. Ideally, we would have been able to study the entire health workforce – including both professionally trained personnel (e.g. nurses) as well as lay health workers – across the globe. We do not intend to present the physician workforce as the only means to achieving health-related goals. For example, systematic reviews of MCH health worker programs [15], as well as literature on task-shifting (the delegation of higher order healthcare tasks to workers with lower training) [16, 17] indicate that healthcare delivery by those with less than physician-level training is not only feasible, but at times, preferred by recipients of care. However, each of these studies and reviews note issues with these modalities of care delivery. Additionally, progress towards MDGs was likely hindered in many countries due to internal instability, inefficient bureaucratic and healthcare systems, and resource depletion and mismanagement, as observed, for example, in Nigeria [18]. Truly, there are both other options for healthcare delivery than simply expecting an increase in PDL; and there are other problems to address as well. However, at the conclusion of this study, we believe we have demonstrated that, regardless of the complex realities of healthcare delivery in the developing world, the ratio of physicians-to-population matters for outcomes. We believe this to be true whether one views PDL as a direct measurement of a contributing factor to health outcomes, or whether PDL is viewed as a proxy indicator for the ‘health’ of the healthcare system in each country.

### Limitations

There are several limitations to this study. The primary limitation that must be recognized is the fact that we performed a relatively simplistic study, utilizing a small set of publically available data points. The exact nature of the relationship between the physician workforce and the achievement of MDGs in any one country is doubtlessly affected by a large number of context-specific factors, as well as by factors which may not be context specific, but for which we did not have a good source of data, such as a systematic record, across many nations, of other health worker density levels. We are also not the first study [11] to note the relationship between the health workforce (and particularly physician density) and MDG outcomes. However, we do offer this report as iterative and new evidence that the relationship is real and must be taken into account in future planning efforts for developmental goals going forward.

## Conclusions

Regardless of the limitations, we are confident that this paper demonstrates that health workforce issues must be taken into account when formulating any set of development goals. Planning across international borders is required both to support the strengthening of health workforce education systems within low and middle income countries, as well as to mitigate the effects of the outflow of medically-trained individuals from poorer nations to richer ones. The current study does not offer any final answers as to how this should be achieved, but it does underscore the importance of an infrastructural and long-term view of health workforce issues in developing countries.
